# Severe septic shock and cardiac arrest in a patient with *Vibrio metschnikovii*: a case report

**DOI:** 10.1186/1752-1947-8-348

**Published:** 2014-10-20

**Authors:** Joakim Jensen, Marlene Ersgaard Jellinge

**Affiliations:** 1 Department of Orthopedic Surgery, Sydvestjysk Sygehus Esbjerg (Southwest Jutland Hospital, Finsensgade 35, DK-6700 Esbjerg, Denmark; 2 Department of Anesthesiology, Sydvestjysk Sygehus Esbjerg (Southwest Jutland Hospital), Finsensgade 35, DK-6700 Esbjerg, Denmark

**Keywords:** Intensive care, Rare condition, Severe sepsis, *Vibrio*

## Abstract

**Introduction:**

*Vibrio metschnikovii* is a very rare species and can be fatal to patients with massive comorbidity. Until now only eight other cases have been reported.

**Case presentation:**

This case report describes a 78-year-old Danish man who presented with fever, hypotension and unconsciousness and he developed cardiac arrest. *Vibrio metschnikovii* was identified in all his blood samples and effective antibiotics were initiated.

**Conclusions:**

The human sources are believed to include shrimps, birds, water, sewage and seafood. We report the first case of *Vibrio metschnikovii* from a Nordic country and the report shows that even though isolation of *Vibrio metschnikovii* from human clinical samples is very rare, it still infects humans and may be fatal, despite sufficient treatment.

## Introduction

*Vibrio metschnikovii* is a Gram-negative, catalase-positive bacillus. Compared to other *Vibrio* species it has a specific biochemical profile characterized by negative oxidase reaction and negative nitrate reduction. Non-human sources are known to include shrimps, crabs, birds, water, sewage and seafood [1].

Severe human infections with *Vibrio metschnikovii* were not described in medical literature until Jean-Jacques *et al*. published a case of bacteremia in a patient with cholecystitis in 1981 [2].

When doing a thorough search in PubMed for *Vibrio metschnikovii* infections, we found eight other cases published.

*Vibrio metschnikovii* cytolysin was purified and characterized in 1988 by Miyake *et al.*[3]. These bacterial strains were isolated from a stool sample taken from a 60-year-old woman admitted with diarrhea and general languor who was diagnosed with a hepatoma and diabetes mellitus during admission. The cytolysin showed hemolytic properties in several animal species.

We describe a patient with severe septic shock and cardiac arrest, caused by *Vibrio metschnikovii*, with multiple comorbidities. The following report shows that even though isolation of *Vibrio metschnikovii* from human clinical samples is very rare, it still infects humans and may be fatal despite sufficient treatment according to international guidelines.

## Case presentation

The patient was a 78-year-old Danish man with 24 hours’ duration of pain, swelling and redness of his left dorsalis pedis. He had fever and was confused. His previous history included gastroenteritis after having shrimps with his wife. She also had gastroenteritis. The general practitioner prescribed antibiotics but after initiating medication the patient became unconscious and his wife called for emergency assistance. His past medical history included bipolar disorder, multiple myeloma, osteoporosis, earlier pituitary tumor and development of frontotemporal dementia. On physical examination, his Glasgow Coma Score (GCS) was 3, blood pressure was 79/38mmHg, heart rate was 115 beats/minute and oxygen saturation was 60%. His respiratory rate was 40 breaths/minute and his temperature was 39°C. In the ambulance he was treated with metaoxedrine, epinephrine, clemastine, oxygen and isotonic sodium chloride. When entering our emergency room he was awake and had GCS 9, blood pressure 87/43mmHg, pulse 110 beats/minute, oxygen saturation 90% and respiratory rate 35 breaths/minute. Blood samples were initially collected from a vein using two aerobic and one anaerobic BacT/ALERT® plastic collection bottles at the time of arrival at the hospital (6 February 2014 9.00 a.m.). After that he was treated with methylprednisolone, ciprofloxacin and meropenem. Arterial blood gas showed a pH 7.11, lactate 4.8mmol/L, oxygen partial pressure arterial 11.9kPa, carbon dioxide partial pressure arterial 3.4kPa, bicarbonate 17mmol/L and hemoglobin 4.2mmol/L. The plastic collection bottles were analyzed in a BacT/ALERT® 3D system (colorimetric technology, bioMérieux), 35°C.

He was transferred to our acute medical unit and on arrival he rapidly went hypotensive again and developed a cardiac arrest. Cardiopulmonary resuscitation was performed, epinephrine was administered and return of spontaneous circulation was observed after 2 minutes. Sedation and intubation was performed and he was transferred to the Intensive Care Unit (ICU). A nurse observed diffuse redness on his left foot, high temperature, petechiae and pain. His right lower leg had petechiae too and minor edema. C-reactive protein (CRP) was 175mg/L, white blood cells 1.0×10^9^/L, platelets 56×10^9^/L and the treatment with meropenem and ciprofloxacin continued combined with metronidazole and fluconazole.

A cardiac echo revealed ejection fraction >55%, a minor mitral valve deficiency and a well-preserved left ventricle. Treatment with inotropic agents was initiated, using norepinephrine because of volume-resistant hypotension and decreased urine output.

The next day an orthopedic surgeon assessed the patient and took him to our operating room to perform acute surgery; the patient’s leg was suspicious of necrotizing fasciitis. On his left foot there was redness, bullae, cyanotic toes and there was no palpable pulse in the arteria dorsalis pedis. Microscopic examination conducted in our laboratory of clinical microbiology found Gram-negative bacteria after Gram-stained smears in all samples from the patient. The treatment with meropenem and metronidazole continued. His CRP was 284mg/L.

The next day our laboratory of clinical microbiology identified *Vibrio metschnikovii* in all his blood samples using matrix-assisted laser desorption/ionization time-of-flight mass spectrometry. There was a 99.9% match score by Basic Local Alignment Search Tool that the bacillus was *Vibrio Metschnikovii*. The result was confirmed by polymerase chain reaction and DNA analysis with 500 base pairs using the FAST MicroSeq® 500 16S rDNA Bacterial Identification System kit (Applied Biosystems). They suggested treatment with meropenem and piperacillin/tazobactam since the antibiotic susceptibility tests found the bacillus to be sensitive to these antibiotics. The bacillus was resistant to ampicillin. His CRP was 318mg/L. He went to surgery and wound necrosis was found on top of vital tissue. Treatment with ciprofloxacin was cancelled and the next step was either treatment with vacuum-assisted closure (VAC) system or in worst case amputation. His CRP was 262mg/L.

The following days his CRP decreased, this urine output increased and at that time the only antibiotic he was taking was piperacillin/tazobactam. The wound healing improved day by day, but he remained somnolent and needed mechanical ventilation. A tracheostomy was established 13 days after admission and he slowly improved during the following days of weaning from the ventilator. During this period he underwent surgical revision twice. Piperacillin/tazobactam was discontinued and the tracheostomy was replaced with an uncuffed tube as he now could manage spontaneous respiration. He was transferred from ICU to our medical department 20 days after admission to our hospital. A VAC system was established on his left foot and split-skin transplantation was performed 5 weeks after admission to our hospital. He was still in hospital 3 months after admission.

## Discussion

Human infections with *Vibrio metschnikovii* are rare. Few cases have been reported and only one case of an infected leg as in our patient [4]. Until now, none of the cases have been from a Nordic country. Our case report reveals a very long stay in hospital until recovery compared with other case reports from patients infected with *Vibrio metschnikovii*[2,4-6].

Infections with *Vibrio metschnikovii* can be fatal as well, especially to a weak patient. Our patient had septic shock and developed cardiac arrest. Hansen *et al.* described a case in 1993 [7] involving a man aged 70 who was a chronic multimorbid (alcoholic cirrhosis, renal insufficiency, diabetes) and admitted with abdominal pains and signs of gastroenteritis with diarrhea and vomiting. A chest X-ray revealed pulmonary infection and blood cultures grew *Vibrio metschnikovii*. This patient’s condition deteriorated and he died of myocardial infarction 5 days after admission.

Another case described by Hansen *et al.*[7] involved an 82-year-old woman admitted with sepsis, respiratory problems and infected chronic leg ulcerations. She was known to have asthma, cardiac insufficiency and emphysema among others. In this case one of three blood cultures grew *Vibrio metschnikovii* and swabs from her leg ulcerations grew a mixed flora including *Vibrio metschnikovii*. This patient was treated with antibiotics, pleurocentesis (bacteriologically sterile) and surgical debridement of the leg ulcerations. She was discharged after almost 4 weeks. In our case report, three of three blood cultures grew *Vibrio metschnikovii* and our patient was almost the same age and had massive comorbidity as well. He did not respond as sufficiently to the treatment as the mentioned woman.

Hardardottir *et al.*[8] published another case of *Vibrio metschnikovii* bacteremia in 1994. An 83-year-old woman was admitted on suspicion of a heart attack, this was ruled out but due to her condition with malaise, chills and high fever, sepsis was suspected. Blood cultures grew *Vibrio metschnikovii* and *Staphylococcus hominis*, one culture also grew *Escherichia coli*.

In 2004 Linde *et al.*[4] published a case of postoperative wound infection in a 64-year-old man after saphenectomy on both legs, with only one side being infected. Healing was achieved with intense topical wound care (no surgery). The treatment in the case of our patient was not that effective and he needed surgery to save his leg. A year later Wallet *et al.*[5] describe a case of *Vibrio metschnikovii* pneumonia. This patient, a 64-year-old man, with known chronic obstructive pulmonary disease, was admitted to ICU with acute respiratory failure. Blood cultures were negative, but bronchial aspirate grew *Vibrio metschnikovii*. The patient underwent 10 days of antibiotics and total admittance time was 15 days. The patient in our case report also needed intensive care treatment and is still in hospital more than 3 months after admission.

*Vibrio metschnikovii* has been seen in children but it is rare. Prasad and Kharidehal [9] described in 2006 the first case, specifically in a baby boy, 5 days after birth. The child was admitted to the Neonatal ICU because of bleeding from the umbilical stump, refusal of feeds and decreased activity for a day. Sepsis was suspected and antibiotics administrated after blood cultures were taken. Blood cultures grew *Vibrio metschnikovii*. The mother was not ill and had no symptoms; cultures were taken from her and were negative.

## Conclusions

We believe that our patient’s meal of shrimps could be the cause of the severe infection described in this case report. In patients who develop *Vibrio* infections, the medical care depends on the clinical presentation and, as in this case, underlying medical conditions. Wound infections can spread to sepsis and suggestion for treatment might be, as in our case, immediate initiation of effective antibiotics, intensive care therapy with aggressive fluid replacement and vasopressor drugs for hypotension. Early debridement of the infected wound may play an important role in successful therapy and thereby avoid amputations and skin graft in the recovery phase. In the case described here the patient’s underlying condition contributed to his long recovery period.

## Consent

Written informed consent was obtained from the patient for publication of this case report and accompanying images. A copy of the written consent is available for review by the Editor-in-Chief of this journal.

## Abbreviations

CRP: C-reactive protein, an acute phase protein synthesized by the liver; GCS: Glasgow Coma Score, a scale for measuring level of consciousness and the resulting points give a patient a score between 3 indicating deep unconsciousness and 15 indicating no impairment; ICU: Intensive Care Unit, a special department of a hospital that provides treatment for critically ill patients; VAC: Vacuum-assisted closure, negative pressure wound therapy as an aid to wound healing.

## Competing interests

The authors have no competing interests, did not receive any financial support and the report has not been presented at any meeting.

## Authors’ contributions

JJ wrote the introduction and was responsible for references. MEJ wrote the case report and was a major contributor in writing the manuscript. The discussion and conclusion sections were written by both authors. Both authors read and approved the final manuscript.
